# Decline in changing Montreal Cognitive Assessment (MoCA) scores is associated with post-stroke cognitive decline determined by a formal neuropsychological evaluation

**DOI:** 10.1371/journal.pone.0173291

**Published:** 2017-03-27

**Authors:** Hui Hui Tan, Jing Xu, Hock Luen Teoh, Bernard Poon-Lap Chan, Raymond Chee Seong Seet, Narayanaswarmy Venketasubramanian, Vijay Kumar Sharma, Christopher Li-Hsian Chen, YanHong Dong

**Affiliations:** 1 Division of Neurology, Department of Medicine, Yong Loo Lin School of Medicine, National University Health System, Singapore; 2 Singapore Clinical Research Institute, Singapore; 3 Memory Ageing and Cognition Center, Department of Pharmacology, Yong Loo Lin School of Medicine, National University Health System, Singapore; 4 Neuroscience Centre, Raffles Hospital, Singapore; 5 Centre for Healthy Brain Ageing (CHeBA) and Dementia Collaborative Research Centre – Assessment and Better Care, School of Psychiatry, UNSW Medicine, The University of New South Wales, Sydney, New South Wales, Australia; Ehime University Graduate School of Medicine, JAPAN

## Abstract

**Objectives:**

We aimed to examine changes in the Montreal Cognitive Assessment (MoCA) and Mini-Mental State Examination (MMSE) scores within a one-year period after stroke/transient ischemic attack (TIA) in associating cognitive decline determined by a formal neuropsychological test battery.

**Methods:**

Patients with ischemic stroke/TIA received MoCA and MMSE at baseline within 14 days after stroke/TIA, at 3–6 months and 1-year follow-ups. The scores of MoCA and MMSE were considered to have declined if there were a reduction of ≥2 points in the respective scores measured across two time points. The decline in neuropsychological diagnosis transitional status was defined by a category transition from no cognitive impairment or any cognitive impairment to a more severe cognitive impairment or dementia.

**Results:**

275 patients with a mean age of 59.8 ± 11.6 years, and education of 7.7 ± 4.3 years completed all the assessments at baseline, 3–6 months and 1-year follow-ups. A decline in MoCA scores from 3–6 months to 1 year was associated with higher risk of decline in diagnosis transitional status (odd ratio = 3.21, p = 0.004) in the same time period whereas there was no association with a decline in MMSE scores.

**Conclusions:**

The decline in MoCA scores from 3–6 months to 1 year after stroke/TIA has three times higher risk for decline in the diagnosis transitional status. The decline of MoCA scores (reduction ≥ 2points) is associated with the decline in neuropsychological diagnosis transitional status.

## Introduction

Cognitive deficits are common after stroke with 10% of patients developing dementia after their first-ever stroke and more than 30% had dementia after recurrent stroke [1]. Post-stroke dementia includes vascular dementia (VaD) among others. Vascular cognitive impairment with no dementia (VCIND) is even more prevalent in post-stroke patients, with a reported rate of 47.7% [2]. Both VCIND and VaD are considered part of the spectrum of vascular cognitive impairment (VCI) [3]. The prognosis of VCI is poor, with higher mortality rate and poorer functional outcome as compared to patients without cognitive impairment [4]. Furthermore, the severity of VCIND differentially predicts survival such that moderate VCIND patients are 3–4 times more likely to die than mild VCIND patients and those without cognitive impairment within the follow-up period of 5 years [4].

Cognitive screening using brief tests is therefore essential to detect VCI and more importantly, decline in brief cognitive test scores over time may be useful in approximating deterioration in cognitive decline as diagnosed by a formal neuropsychological test battery. Thus changing cognitive test scores may be a timesaving measures to aid cognitive recovery with appropriate rehabilitation to prevent further deterioration. The Mini-Mental State Examination (MMSE) is one of the most widely used cognitive screening instruments [5]. However, it has been criticized for inadequate criterion validity especially in detecting VCI due to lack of frontal-executive function test items [6]. The Montreal Cognitive Assessment (MoCA) is a relatively new screening instrument developed to detect mild cognitive impairment [7]. By including frontal-executive function items such as trail test, clock drawing and copying a cube as well as abstract reasoning such as similarities test, MoCA may be relatively more sensitive to detect characteristic cognitive deficits in VCI. The superiority of MoCA over MMSE in detecting cognitive impairment is debatable as findings from previous studies are mixed, with some studies recommending the MoCA [6] while others reported that both instruments are equivalent [8, 9].

Few studies have evaluated the abilities of MMSE and MoCA scores within two weeks after stroke event (sub-acute stroke phase) for predicting cognitive impairment at chronic stroke phase. To date, two studies reported that the MMSE and MoCA were both predictive of cognitive impairment determined by formal neuropsychological assessments at 3–6 months after the index cerebrovascular event [10, 11]. Recently, the changing scores of MoCA, through multiple assessments over 90 days after stroke event have been reported to map the temporal patterns of transient cognitive deficits [12]. However, the utility of a decline in brief screening test scores through consecutive assessments over time in suggesting deterioration of neuropsychological diagnosis is unknown and should be investigated. Therefore, our primary aim was to examine the association of declining MoCA and MMSE test scores from sub-acute stroke phase to chronic stroke phase, with the decline in neuropsychological diagnosis from 3–6 months after stroke to a year later.

## Methods

### Participants

This study uses the same methods and participant population reported previously [9, 10]. Briefly, 904 patients admitted to the National University Health System were screened for eligibility based on the inclusion criteria and recruited 400 patients (≥ 21 years old) in the sub-acute stroke phase, i.e., within 2 weeks after ischemic stroke/transient ischemic attack (TIA). Patients were excluded if they have major physical disability (modified Rankin scale (mRS) > 4), significant aphasia/dysarthria (National Health Stroke Sore (NIHSS) best language (Aphasia) and dysarthria score > 1), active psychiatric disorder and pre-existing dementia based on a score of > 3.38 on the Informant Questionnaire on Cognitive Decline in the Elderly (IQCODE) [13, 14].

Written informed consent was obtained from eligible patients after they were assessed by a healthcare professional on their capacity to consent. Otherwise, proxy consent was obtained from legally acceptable representatives, if the patients were assessed to not have the capacity to give the consent.

### Measures

The MMSE and MoCA were administered by trained research psychologists at baseline (within two weeks of index cerebrovascular event), at 3–6 months and 1-year after stroke event. A dichotomous MMSE change category variable defined “decline” as a reduction of ≥ 2 points in MMSE total scores over two consecutive time points and “no decline” for other score changes [15]. A dichotomous MoCA change category variable also defined, “decline” as a reduction of ≥ 2 points in MoCA total scores over two consecutive time points based on the reliability change index cutoff of ±1.73 [16] and “no decline” for other score changes. These research psychologists administering the MMSE and MoCA tests were blinded to the neuropsychological diagnosis at 3–6 months and 1-year follow-ups.

Trained research psychologists also administered a formal neuropsychological test battery previously validated for Singapore and were commonly used in stroke clinical research [17] at 3–6 months and 1-year follow-ups. The domains of the formal neuropsychological battery included verbal and visual memory, attention, language, visuomotor speed, visuoconstruction and executive function. It takes about 1.5 hours to 2 hours to administer this battery.

This formal neuropsychological test battery diagnosed patients by categorizing them as having no cognitive impairment (NCI), VCIND mild (defined as impairment in ≤2 cognitive domains), and VCIND moderate (defined as impairment in ≥3 cognitive domains) [10]. The outcome measure of this study is the neuropsychological diagnosis transitional status, which indicates whether the patient has “decline” or “no decline” status between two consecutive neuropsychological assessments. The “decline” status is defined by either a category transition (a) from NCI to any cognitive impairment (defined as ≥1 cognitive domains impaired), (b) from VCIND mild to VCIND moderate, and dementia (functional loss associated with cognitive impairment according to DSM-IV criteria), and (c) VCIND moderate to dementia. Other category transitions including improvement in neuropsychological diagnosis (e.g., from VCIND mild to NCI) were defined as “no decline” status in this neuropsychological diagnosis transitional status variable.

The mood of patients was assessed with the 15-item Geriatric Depression Scale (GDS) at 3–6 months and 1-year follow-ups.

### Statistical analysis

All data analyses were conducted using the statistical package R version 3.2.0 for Windows. Between-group comparisons were performed using either independent sample t-tests or Mann-Whitney U tests for quantitative variables and Pearson’s chi-square for categorical variables. Logistic regressions were conducted to determine the factors that were associated with the diagnosis transitional status (decline vs. no decline).

## Results

**Fig 1:**
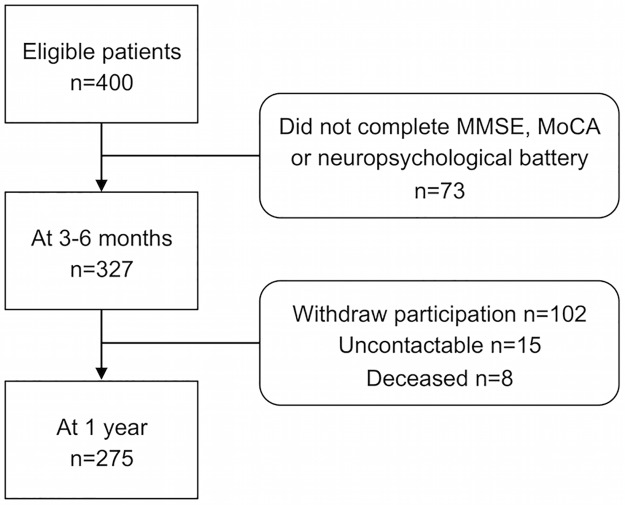
Flow diagram of participation.

At baseline, there were 400 patients, of which most were Chinese (70.3%), male (69.8%) with a mean age of 59.8 ± 11.6 years, and education of 7.7± 4.3 years. At 3–6 months after stroke/TIA, 327 patients completed MMSE, MoCA and the formal neuropsychological battery. At 1 year, 275 patients completed the same 3 cognitive measures. The major reasons for the attrition were that the patients withdrew their participation (n = 102), were un-contactable (n = 15) or deceased (n = 8). A comparison of the demographic characteristics, baseline MMSE and baseline MoCA scores between participants who completed the assessments and those who dropped out from this study showed that there were no significant differences. A flow diagram of study participation is shown in Fig 1. The most prevalent type of impairments was found in the visuomotor speed domain at both 3–6 months and 1 year. The patients had median GDS scores of 2 at both 3–6 months and 1 year indicating no self-reported depression symptoms.

At 3–6 months after stroke/TIA, 33 (12%) patients showed decline in their MMSE scores and 39 (14.2%) had declined in their MoCA scores from their respective baseline scores. From 3–6 months to 1-year, 65 (23.6%) patients had declined in their MMSE scores, 85 (30.9%) had declined in their MoCA scores, and 31 (11.3%) had declined in their diagnosis transitional status. The characteristics of patients classified by their diagnosis transitional status are shown in Table 1.

**Table 1:** Patient characteristics.

	Diagnosis Transitional Status				*p*
	Decline* (*n* = 31)		No Decline** (*n* = 244)		
Age, mean (SD)	62.00	(10.81)	59.45	(11.44)	0.21
Gender, female (%)	6.00	(19%)	75.00	(31%)	0.27
Education, mean (SD)	6.97	(4.43)	7.83	(4.32)	0.25
Ethnicity					0.08
Chinese (%)	21.00	(68%)	177.00	(73%)	
Malay (%)	4.00	(13%)	49.00	(20%)	
Indian (%)	6.00	(19%)	16.00	(7%)	
Others (%)	0.00	(0%)	2.00	(1%)	
MMSE					
Baseline score, mean (SD)	24.55	(3.20)	24.73	(3.90)	0.44
^#^Decline—from baseline to 3–6 months (%)	4.00	(13%)	29.00	(12%)	1.00
^#^Decline—from 3–6 months to 1-year (%)	8.00	(26%)	57.00	(23%)	0.94
MoCA					
Baseline score, mean (SD)	20.29	(4.12)	20.96	(5.31)	0.27
^Decline—from baseline to 3–6 months (%)	4.00	(13%)	35.00	(14%)	1.00
^Decline—from 3–6 months to 1-year (%)	17.00	(55%)	68.00	(28%)	<0.01

*Decline = decline in diagnosis transitional status from 3–6 months to 1-year;

**No Decline = stable or improve in diagnosis transitional status from 3–6 months to 1-year;

^#^Decline = decline status in MMSE change category;

^Decline = decline status in MoCA change category;

*p* = p-value; SD = standard deviation; MMSE = Mini-Mental State Examination; MoCA = Montreal Cognitive Assessment

There were no significant differences between the diagnosis transitional status for the “decline” in MMSE change category from baseline to 3–6 months, and from 3–6 months to 1 year, and for the “decline” in MoCA change category from baseline to 3–6 months. From 3–6 months to 1 year after stroke/TIA, a significant difference was found between the “decline” in diagnosis transitional status and “decline” in MoCA change category (*p* < 0.01). More than half of the patients (55%) who had declined in their diagnosis transitional status showed a decline in their MoCA change category.

Using continuous MoCA scores with MoCA baseline scores and change in MoCA scores from baseline to 3–6 months as covariates, a one-point decrease in MoCA scores from 3–6 months to 1-year increased the odds of having a “decline” in the diagnosis transitional status by 16% (OR = 1.16, 95% CI: 1.01–1.34, *p* = 0.04). Using the MoCA change category with the MoCA baseline scores and change in MoCA scores from baseline to 3–6 months as covariates, the result showed that patients with a decline in MoCA scores from 3–6 months to 1 year was associated with higher risks of a decline in the diagnosis transitional status (OR = 3.21, 95% CI: 145–7.08, *p*<0.01).

## Discussion

The principal finding of this study is that the decline in MoCA scores is significantly associated with having a decline in diagnosis transitional status. Patients with a decline in MoCA scores from 3–6 months to 1-year are three times more likely to have deteriorated in their diagnosis transitional status. In comparison, there is no significant association between the decline in MMSE scores and having a decline in diagnosis transitional status from 3–6 months to 1-year after stroke/TIA. This result suggests that the MMSE may not be as sensitive as MoCA in detecting changes in cognition. With its superior sensitivity, the MoCA may be more useful than the MMSE clinically for monitoring cognition over time to implement early intervention for high-risk patients and to customize rehabilitation for patients with post-stroke VCI. A decline in MoCA test scores, defined as a reduction of ≥ 2 points over two consecutive screenings from 3–6 months to 1 year, can potentially be a practical and time-efficient method to inform clinicians that their patients warrant further neuropsychological examinations.

Few studies have examined the utility of changing scores in repeated brief cognitive tests in post-stroke VCI. A recent study reported that serial MoCA tests mapped the temporal patterns of transient cognitive deficits [12]. However, the drawback of this study is the lack of gold standard formal neuropsychological assessments over time. To the best of our knowledge, this is the first study that has examined the temporal decline in brief cognitive tests (MoCA and MMSE) against the deterioration in diagnosis determined by serial neuropsychological assessments.

No significant associations were found between the decline in the diagnosis transitional status and a decline in the scores of the MoCA and MMSE from baseline to 3–6 months. This could be due to spontaneous recovery in cognition (e.g., language) [18] as a result of the short timeframe of 3–6 months between the two assessments.

Several limitations of this study should be acknowledged. First, our results may not be generalizable to all stroke populations, as most of the patients were young, and had milder stroke with minimal disability. Second, the sample size for patients with decline in neuropsychological diagnosis status is too small to examine the reduction of MoCA scores between mild and moderate VCIND groups, as well as those with dementia. Third, the follow-up time frame of 1-year is relatively short and the number of serial assessment is two only, which may limit tracking cognition over time. Third, it would be ideal and more reliable to establish diagnosis with a formal neuropsychological evaluation at baseline. However, this is not practical in clinical practice due to fluctuation in cognitive functioning in the subacute stroke phase. Future studies should examine the association of the decline in MoCA scores in a larger cohort of stroke/TIA patients over a longer follow-up duration (≥3 years) with the deterioration in neuropsychological status.

## Conclusion

The decline in MoCA scores from 3–6 months to 1 year after stroke/TIA has three times higher risk for decline in the diagnosis transitional status determined by neuropsychological test performance. This significant association between the decline in MoCA and the decline in the diagnosis transitional status suggests that MoCA is superior to MMSE in detecting cognition changes over time for patients with VCI. This finding also suggests that using MoCA to monitor the change in cognition of patients with VCI may be practical in clinical setting.
